# Comparison of the *in vitro* anthelmintic effects of *Acacia nilotica* and *Acacia raddiana*

**DOI:** 10.1051/parasite/2017044

**Published:** 2017-11-27

**Authors:** Geneviève Zabré, Adama Kaboré, Balé Bayala, Luciana M. Katiki, Lívio Martins Costa-Júnior, Hamidou H. Tamboura, Adrien M.G. Belem, Adibe L. Abdalla, Vincent Niderkorn, Hervé Hoste, Helder Louvandini

**Affiliations:** a Laboratoire de Biologie et Santé Animales-DPA/INERA, 04 BP 8645 Ouagadougou 04 Burkina Faso; b Université de Ouagadougou / UFR-SVT, 03 BP 7021 Ouagadougou 03 Burkina Faso; c Instituto de Zootecnia (SAA, APTA), Rua Heitor Penteado 56, Nova Odessa, SP, cep 13460-000 Brazil; d Universidade Federal do Maranhão − UFMA, Campus do Bacanga CEP 65080-805 São Luís- MA Brazil; e Université Nazi Boni, Bobo-Dioulasso, 01 BP 3770 Ouagadougou 01 Burkina Faso; f Universidade de São Paulo, Centro de Energia Nuclear na Agricultura, NAPTSA, CP 96, CEP 13.400-970, Piracicaba, SP Brazil; g UMR1213 Herbivores, INRA − Clermont-Ferrand, Vetagro Sup, 63122 Saint-Genès-Champanelle France; h UMR IHAP 1225 INRA/ENVT, 23 Chemin des Capelles, 31076 Toulouse Cedex France

**Keywords:** Natural products, tannins, secondary plant metabolites, nematodes, anthelmintics

## Abstract

Gastrointestinal nematodes are a major threat to small ruminant rearing in the Sahel area, where farmers traditionally use bioactive plants to control these worms, including *Acacia nilotica* and *Acacia raddiana*. The main aim of this study was to screen the potential anthelmintic properties of aqueous and acetone extracts of leaves of these two plants based on three *in vitro* assays: (1) the egg hatch inhibition assay (EHA); (2) the larvae exsheathment inhibition assay (LEIA) using *Haemonchus contortus* as a model; and (3) an adult mortality test (AMT) applied on *Caenorhabditis elegans*. For the EHA, only *A. raddiana* was effective with IC_50_ = 1.58 mg/mL for aqueous extract, and IC_50_ = 0.58 mg/mL for acetonic extract. For the LEIA, all extracts inhibited the exsheathment of larvae compared to the controls, and the aqueous extract of *A. nilotica* was more larvicidal with IC_50_ = 0.195 mg/mL. In general, all responses to the substances were dose-dependent and were significantly different from the control group (*p* < 0.05). For the AMT, the extracts of the two *Acacia* species were effective but *A. raddiana* showed greater efficacy with 100% mortality at 2.5 mg/mL and LC_50_ = 0.84 mg/mL (acetonic extract). The addition of polyvinyl polypyrrolidone (PVPP) to the extracts suggested that tannins were responsible for blocking egg eclosion and inducing adult mortality but were not responsible for exsheathment inhibition. These results suggest that the leaves of these *Acacia* species possess ovicidal and larvicidal activities *in vitro* against *H. contortus*, and adulticidal effects against *C. elegans*.

## Abstract (fr)

Les nématodes gastro-intestinaux demeurent une menace majeure de l'élevage des petits ruminants en zone sahélienne, où les éleveurs utilisent traditionnellement des plantes bioactives pour maîtriser ces parasites, notamment *Acacia nilotica* et *Acacia raddiana*. L'objectif de ce travail était de vérifier l'efficacité anthelminthique d'extraits aqueux et acétoniques de feuilles de ces deux plantes sur la base de 3 tests *in vitro *: 1) l'inhibition de l'éclosion des œufs (EHA), 2) l'inhibition du dégainement des larves (LEA) appliquée au modèle *Haemonchus contortus* et 3) la mortalité des *Caenorhabditis elegans* adultes (AMT). Pour EHA, seul *A. raddiana* a été efficace avec IC_50_ = 1,58 mg/mL pour l'extrait aqueux et IC_50_ = 0,58 mg/mL pour l'extrait acétonique. Pour LEA, tous les extraits ont inhibé le dégainement des larves par rapport aux témoins et l'extrait aqueux d'*A. nilotica* a été plus larvicide avec un IC_50_ = 0,195 mg/mL. De façon générale, toutes les concentrations agissent de façon dose-dépendante et sont significativement différentes du groupe témoin (P < 0,05). Pour AMT, les extraits des deux *Acacia* ont été efficaces mais *A. raddiana* a été plus efficace avec 100% de mortalité à 2,5 mg/mL avec une DL_50_ = 0,84 mg/mL (extrait acétonique). L'addition de polyvinylpolypyrrolidone (PVPP) aux extraits a montré que les tannins seraient responsables de l'inhibition de l'éclosion des œufs et de la mortalité chez les vers adultes, mais ne seraient pas responsables de l'inhibition du dégainement des larves. Ces résultats suggèrent que les feuilles de ces deux *Acacia* possèdent des activités ovicides et larvicides *in vitro* contre *H. contortus* et des effets sur les *C. elegans* adultes.

## Introduction

Small ruminant rearing contributes strongly to human livelihoods in developing countries, especially for rural farmers [65]. In Sahelian countries, such as Burkina Faso, Mali and Niger, livestock play an important role in the economy [5]. In Burkina Faso, the livestock sector alone contributes over USD 51.3 million annually to the national economy, of which 32% is from small ruminants [49]. Despite the importance of this sector, small ruminant rearing faces feeding and health constraints that limit animal production. Among the health constraints, gastrointestinal nematodes (GINs), and *Haemonchus contortus* in particular (a highly prevalent and pathogenic species of GIN [15,56]), lead to low performances and higher animal mortality [35,58,67]. Like in many developing countries, the majority of livestock owners in rural areas do not use synthetic anthelmintic (AH) drugs because they are not affordable [31,55]. Therefore, they usually rely on practices related to ethnoveterinary medicine to control health problems in livestock [26]. In the Sahel, many natural fodder plants such as different species of *Acacia*, are used to control disease in ruminants, especially GINs. The use of such bioactive plants has several advantages for local farmers: (a) they are locally available; (b) they cost less compared to synthetic AH drugs; and (c) they are well accepted and consumed by sheep and goats.

An ethnoveterinary survey performed in this area of the Sahel showed that among *Acacia* species targeted for ruminants, *Acacia nilotica var adansonii* (Guill. & Perr.) O. Ktze and *Acacia raddiana* (Savi) are commonly used because of their high nutritional value and their potential AH effects. [70]. All of the breeders investigated used *A. raddiana* essentially as feed for cattle. However, 57% and 43% of the breeders questioned the use of *A. nilotica* to treat and to feed the cattle, respectively. For example, decoctions prepared from the pods and dried leaves of *A. nilotica* are used to treat foot-and-mouth disease (98%), diarrhoea (73%), leg ulcers (51%), loss of appetite (36%) and gingivitis (32%) in small ruminants [70]. In Mauritania, the powdered seed of *A. nilotica* macerated in fresh water is used to treat diarrhoea. The fruit, leaves and bark extracts of *A. nilotica* have been examined in *in vitro* studies [11,12,34,45] and their effects confirmed in *in vivo* studies [11,12,46]. However, information on *A. raddiana* is sparse.

Both *A. nilotica* and *A. raddiana* species are assumed to contain tannins [61]. Many *in vitro* and *in vivo* studies have now provided consistent evidence to support the AH effect of feed containing tannins and other polyphenols against abomasal and intestinal parasitic nematodes [27,29]. These previous investigations have shown that some local plants that contain tannins can impair different key biological processes of the parasitic nematode life cycle: (i) the establishment of the infective third-stage larvae [18,20,47]; (ii) the excretion of eggs by adult worms [41,47,66] and (iii) the development of nematode eggs into larvae [51], and can therefore be used as nutraceuticals [30].

The objectives of this study were therefore two-fold: (1) to evaluate, based on *in vitro* assays, the anthelmintic effects of both aqueous and acetonic extracts of the two *Acacia* plants on two different development stages of *H. contortus* and on adult nematode mortality of members of *Caenorhabditis elegans*; (2) to confirm the possible role of tannins and related polyphenols on *in vitro* AH effects using the tannin inhibitor polyvinyl polypyrrolidone (PVPP).

## Materials and methods

### Plant materials

Fresh leaves of *A. nilotica* and *A. raddiana* were collected in December 2014 at Dori (14° and 15° N; 0° and 3° W), located in north-eastern Burkina Faso. The climate of the region, classified as Sahelian, is marked by a long dry season from November to June and a short rainy season from July to September. The specimens were identified by reference to the herbarium of the French National Centre of Scientific and Technological Research in Ouagadougou, Burkina Faso. Harvested leaves were then cleaned with water and dried at room temperature for a week according to the procedure used by traditional healers in the country.

Dried leaves were first transformed into powders using a blade grinder (basic IKA A 11) and the water content was determined according to the Association of Analytical Communities (AOAC) [6]. Two grams of each powder were dried at 105 °C for 3 h and cooled in a desiccator for 30 min before being weighed. The percentage of water content was calculated according to the following formula: % water _content_ = ((powders _before drying_ (g) − powders _after drying_ (g))/powders _before drying_ (g)) x100.

### Preparation of extracts

Two extracts were prepared for each *Acacia* species: one aqueous and one water/acetonic extract. For aqueous extracts, 150 g of each powdered material were macerated with 750 mL of distilled water for 24 h. Macerated extracts were then filtered over cotton wool and concentrated under reduced pressure in a rotary evaporator at 40-50 °C before being stored at 4 °C. For water/acetonic extracts, 150 g of each powder were macerated with 750 mL of mixed acetone/water (70/30) (v/v) for 72 h. Macerated extracts were filtered over Whatman paper before being concentrated under reduced pressure in a rotary evaporator at 40-50 °C and stored at 4 °C. Dry extracts were subjected to phytochemical screening in order to identify the main phytochemical groups.

### Quantification of tannins and polyphenolic compounds

Total tannins (TT), total phenols (TP) and condensed tannins (CT) were determined according to Makkar [39] and Makkar et al. [38]. TP and TT were determined by adding 250 µL of Folin-Ciocalteu reagents (1N) and 1.25 mL sodium carbonate solution (20% Na_2_CO_3_) to an aliquot of the supernatant and then taking absorbance readings at 725 nm. To determine TT, a binding tannin agent, 100 mg of insoluble polyvinyl polypyrrolidone (PVPP), was added to the extract and the measurement was repeated. A calibration curve was prepared from aliquots of the solution of tannic acid. The difference between measurements of TP and TP + PVPP extract readings was an estimate of TT. The concentrations of TP and TT were calculated as tannic acid equivalents (eq) and expressed as g/kg DM. CT were expressed as leucocyanidin equivalents (% of DM) and were determined using 3 mL butanol-HCl reagent and 0.1 mL ferric reagent. The absorbance was read at 550 nm.

### Materials for *in vitro* assays

The faecal matter of a donor goat experimentally infected with *H. contortus* was collected at the Department of Pathology at the Federal University of Maranhão (UFMA) (Brazil) to obtain the eggs and L_3_ larvae of *H. contortus*. *C. elegans* adult nematodes were obtained by culture according to the method of Chitwood and Feldlaufer [22]. All procedures were approved by the Ethics Committee for Animal Experimentation of the Federal University of Maranhão under number 23115018061.

### *In vitro* assays

Three different tests were performed: the egg hatching assay (EHA), the L_3_ larval exsheathment assay (LEIA) of *H. contortus*, and the *C. elegans* adult mortality assay (AMT).

### Egg hatching assay

This assay was performed according to the method described by Coles et al. [23]. The fresh faecal matter was recovered, crushed and filtered four times on 1-mm, 105-µm, 55-µm and 25-µm sieves. Eggs collected from the 25-µm sieve were centrifuged at 450 g /20 °C for 10 min and the supernatant was removed and replaced by an NaCl solution (density: 1.2), mixed and centrifuged at 1012 g /20 °C for 5 min. The recovered supernatant was filtered with a 25-μm sieve, washed and centrifuged three times at 450 g /20 °C for 10 min. The quantity was adjusted to reach a concentration of 100 eggs/mL. Five increasing concentrations of solutions were prepared for each extract: 0.3, 0.6, 1.25, 2.5 and 5.0 mg/mL diluted in PBS (0.1 M phosphate, 0.05 M NaCl, pH 7.2) for the two aqueous extracts, and in 2% methanol for the acetonic extracts. To test the direct effect of the extracts on nematodes, 100 µL of each concentration + 100 µL of eggs were placed in each well (96-well plate). Four replicates were performed per concentration. The plates were covered with Parafilm and incubated at 27 °C for 48 h. After 48 h of incubation, hatching was stopped by adding lugol iodine solution, and the number of L_1_ larvae and eggs per well was counted using a reverse microscope (magnification x 10). Thereafter, the percentage of hatched eggs was determined using the following ratio: number of L_1_/(number of eggs + number of L_1_).

### Larval exsheathment inhibition assay

The assay on larval exsheathment inhibition was performed as previously described by Bahuaud et al. [13]. Larvae were obtained by larval culture from goat faecal matter incubated at room temperature for 14 days to obtain L_3_ larvae at the concentration of 2000 larvae/mL. Five increasing concentrations were prepared for each of the two extracts: 2.4, 1.2, 0.6, 0.3 and 0.15 mg/mL, all diluted in phosphate-buffered saline (PBS) (0.1 M phosphate, 0.05 M NaCl, pH 7.2) for aqueous extracts and 1% methanol for acetonic extracts. PBS and 1% methanol were used as a negative control. Briefly, 1 mL of L_3_ larvae solution was incubated with 1 mL of extract at 20 °C for 3 h, and then washed with PBS and centrifuged three times at 1380 g for 3 min. Then, 200 µL of the larvae were subjected to the artificial exsheathment process by adding 200 µL of a Milton solution (2% w/v sodium hypochlorite and 16.5% sodium chloride) diluted to 1/300 with PBS. Four replicates were performed per concentration. The kinetics of larval exsheathment in the different experimental treatments were then monitored at 0, 20, 40 and 60 min intervals by microscopic observations (40×).

### Mortality assay of adult *C. elegans*

Adult *C. elegans* were isolated according to Katiki et al. [36]. The test was performed with young adults and adults with intact cuticles. Five concentrations were prepared for the assay: 0.6, 1.25, 2.5, 5 and 10 mg/mL and two negative controls: salt solution M-9 (1.5 g KH_2_PO_4_; 3 g Na_2_HPO_4_; 2.5 g NaCl; 0.5 mL 1M MgSO_4_, final volume of 500 mL) for aqueous extracts, and 2% dimethyl sulfoxide (DMSO) for acetonic extracts used as a solvent. The test was performed using 48-well plates and a nematode stock of 50 larvae/100 µL. In each well, nematodes were placed in contact with each concentration to a final volume of 500 µL/well with four replicates. The plates were then incubated at 25 °C for 20 h. After incubation, plates were read using a microscope and all adult worms were counted and determined as dead or alive, according to Skantar et al. [63]. They were considered dead when they did not show any movement and as alive when there were at least some tails, head or pharyngeal movements (during 10 s of observation). The negative control consistently showed 95-100% live adults at 20 h after incubation.

### Verification of the role of tannins by use of PVPP

In order to confirm the role of tannins on the AH effect, another series of EHA, LEIA and AMT was performed with both extracts of each *Acacia* species. However, for EHA, only *A. raddiana* extracts were used for this test because *A. nilotica* was not effective. Thus, the best concentrations of each extract (5 mg/mL + 100 mg of PVPP for EHA, 10 mg/mL + 200 mg of PVPP for AMT and 1.2 mg/mL + 250 mg of PVPP for LEIA) were pre-incubated with PVPP for 2 h, centrifuged at 1012 g / 10 min at 20 °C before being used for the assay. After centrifugation, all supernatants were removed to perform the three assays according to the procedure previously described and the results compared to an assay without the addition of PVPP. The ratio of pre-incubated PVPP was 1/10 for EHA and AMT. The rate for LEIA was 1/40.

### Statistical analyses

Data from parasite tests were recorded on Excel 2010 (Microsoft corporation) and transformed to log (x + 1) before being subjected to a variance analysis with SAS software 2010, Version 6.20.4. IC_50_ and LC_50_ were calculated by probit analysis. IC_50_ and LC_50_ were considered significantly different when the 95% LC fails to overlap [60]. Comparison of averages (expressed as mean ± standard error of mean) was performed using the Kruskal-Wallis test at 0.05.

## Results

### Quantification of tannins and polyphenolic compounds

Table 1 presents the content of total phenol (TP), condensed tannins (CT) and total tannins (TT) for both *Acacia* species. *A. nilotica* was rich in CT compared to *A. raddiana* which was rich in TT and TP.

**Table 1:** Quantity of condensed tannin (CT), total tannin (TT) and total polyphenols (TP) in Acacia species.

Samples	CT	TT	TP
*A. nilotica*	53.60	54.23	76.66
*A. raddiana*	3.41	391.56	401.28

CT: g of leucocyandin/kg dried mass (DM) TT and TP: g of tannic acid/kg DM.

### Egg hatch inhibition assay

Tables 2 and 4 show the percentage of inhibition of egg hatching at different concentrations and 50% inhibition concentration for *H. contortus*. In general, the two controls recorded the lowest percentages of inhibition (4.3 and 9.8%, respectively, for PBS and 2% methanol) and were significantly different for all extracts (*p* < 0.05). The inhibition of egg hatching increased with increasing concentrations. However, *A. nilotica* was not effective and recorded a percentage of inhibition of less than 22% and an IC_50_>5 mg/mL. The percentage of egg inhibition of *A. raddiana* ranged from 12.67% to 92.76% with an IC_50_ = 1.58 mg/mL for the aqueous extract, and from 27.34% to 91.45% for acetonic extract with an IC_50 _= 0.58 mg/mL.

**Table 2:** Percentages of egg hatch of *H. contortus* after 48 h incubation with *Acacia nilotica* and/or *Acacia raddiana* extract at different concentrations.

Concentration (mg/mL)	*A. nilotica*		*A. raddiana*	
		
	Aqueous extract	Acetonic extract	Aqueous extract	Acetonic extract
5	21.14 ± 2.34 a	18.72 ± 3.88 a	92.76 ± 1.92 a	91.45 ± 1.43 a
2.5	15.56 ± 3.34 b	17.23 ± 1.61 a	85.78 ± 2.09 a	88.81 ± 2.53 a
1.25	7.51 ± 1.71 c	10.95 ± 1.24 ab	40.83 ± 2.75 bA	79.88 ± 3.02 abB
0.625	6.48 ± 1.97 c	10.33 ± 1.63 ab	14.47 ± 1.52 cA	51.67 ± 2.83 cB
0.3125	3.42 ± 1.94 dA	9.85 ± 3.42 abB	12.67 ± 3.83 cdA	27.34 ± 2.18 dB
PBS	4.29 ± 2.01 d	-	4.29 ± 2.01 e	-
Methanol 2%	-	9.81 ± 9.98 ab	-	9.81 ± 9.94 e
P	0.0024	0.0067	0.0016	0.0013

**-** : no administered

(a,b,c,d,e) are compared means within the columns and (A,B) indicate differences within the lines (different extracts) for the two *Acacia* species for each plant. Different letters indicate significant differences (*p* < 0.05)

**Table 4:** Inhibitory concentrations (mg/mL) in the EHA and LEIA (mg/mL) with *Haemonchus contortus* (IC_50_) and lethal concentrations (mg/mL) for the AMT with *Caenorhabditis elegans* (LC_50_) with respective 95% confidence intervals for the different plant extracts.

Plant	Extract	Assay		
		
		EHA	LEIA	AMT
*A. nilotica*	Aqueous	> 5	0.195 (0.180 – 0.211)a	1.01 (0.78 - 1.23)a,b
	Acetonic	> 5	0.224 (0.226 -0. 265)b,c	5.39 (4.24 - 7.42)c
*A. raddiana*	Aqueous	1.36 (1.16-1.59)b	0.331 (0.252 – 0.408)c	1.43 (1.07 - 1.84)b
	Acetonic	0.68 (0.51-0.87)a	0.207 (0.173 – 0.246)a,b	0.84 (0.79 - 0.89)a

(a,b,c) are compared between lines (different extracts) for the two *Acacia* species for each plant. Different letters indicate significant differences.

### Larval exsheathment inhibition essay

Both extracts revealed inhibition activity on L_3_ larvae exsheathment of *H. contortus*. The aqueous extract of *A. nilotica* showed the lowest concentration of inhibition (IC_50_) compared to all of the extracts (Table 4).

### Mortality of adult *Caenorhabditis elegans*

Tables 3 and 4 present the efficacy of the extracts on adult *C. elegans* mortality and the lethal concentration. In general, the two controls recorded the lowest mortality rate (4.69% and 0.84%, respectively, for M-9 and 2% DMSO). The differences between concentrations and controls were highly significant (*p* < 0.001). Moreover, both extracts induced adult *C. elegans* mortality. When the concentration increased, the percentage of adult mortality increased only for the acetonic extract of *A*. *raddiana,* which reached 100% mortality at 2.5 mg/mL with the lowest lethal concentrations: LC_50_ = 0.84 mg/mL compared to the other extracts.

**Table 3:** Percentage of adult *Caenorhabditis elegans* mortality after 20 h of incubation with *Acacia nilotica* and *Acacia raddiana* extract at different concentrations.

Concentration (mg/mL)	*A. nilotica*		*A. raddiana*	
		
	Aqueous extract	Acetonic extract	Aqueous extract	Acetonic extract
10	94.97 ± 3.9aA	85.89 ± 3.0 aB	100 ± 0aA	100 ± 0aA
5	87.73 ± 2.9 bA	37.52 ± 2.5 bB	96.75 ± 1.6 aA	100 ± 0aA
2.5	81.78 ± 5.2 bA	19.04 ± 2.2 cB	80.32 ± 8.4 bA	100 ± 0aB
1.25	51.42 ± 2.8cA	3.31 ± 1.2 dB	11.25 ± 1.9 cA	81.08 ± 2.3bB
0.625	34.38 ± 5.5 dA	1.19 ± 2.1 dB	11.16 ± 4.1cA	22.53 ± 1.3cB
M9	4.69 ± 1.8 e	-	4.69 ± 1.8d	-
DMSO 2%	-	0.84 ± 0.8 d	-	0.84 ± 0.8 d
p	< 0.001	< 0.001	< 0.001	< 0.001

**-** : not administered.

(a,b,c,d,e) are compared means in the columns and (AB) in the lines. Different letters indicate significantly values (*p* < 0.05).

### Using the inhibitor PVPP to assess the role of tannins and polyphenols

**Figure 1:**
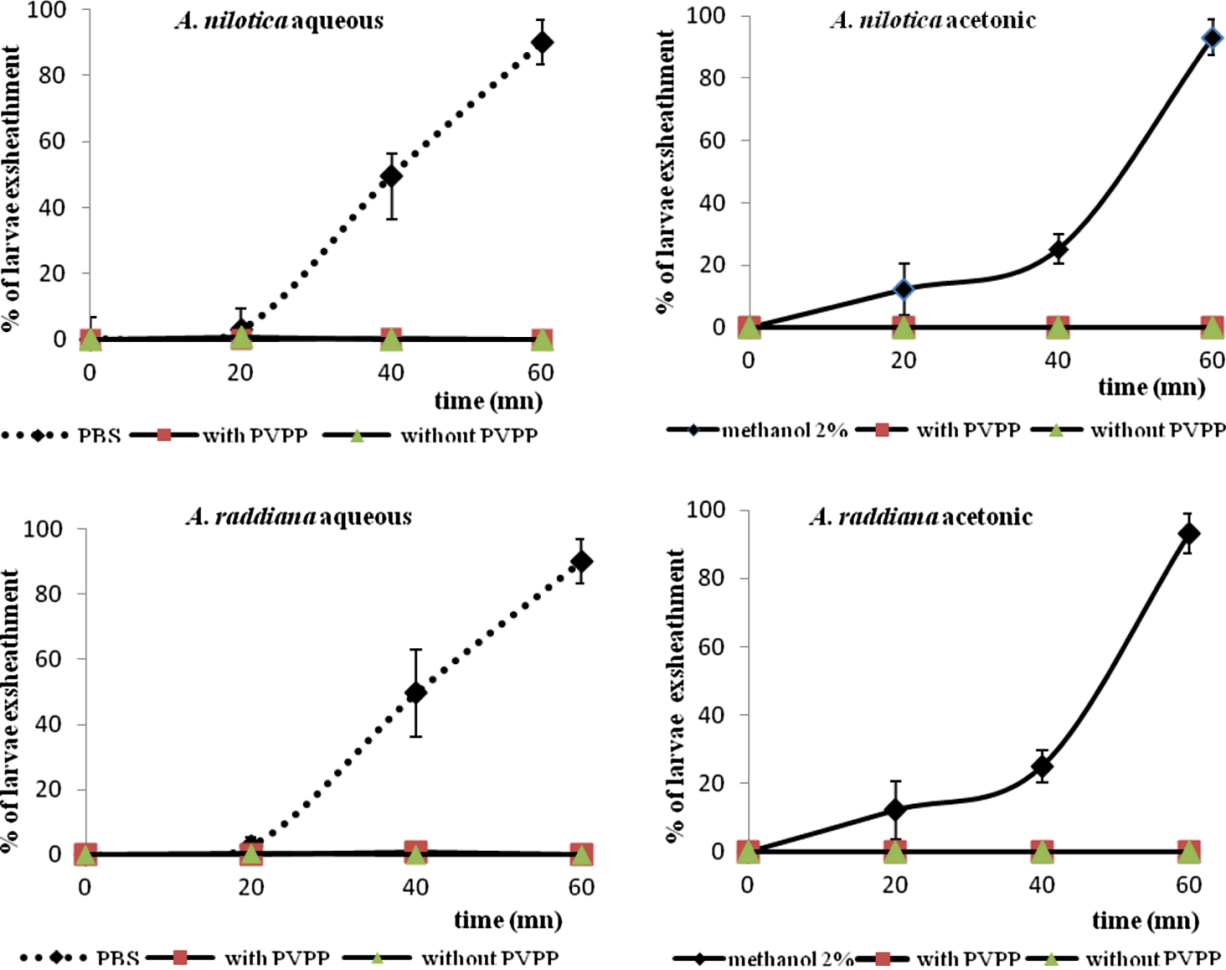
Larval exsheathment of *H. contortus* in the presence of *acacia* extracts at a concentration of 1.2 mg/mL, and its combination with polyvinyl polyvinylpyrrolidone (PVPP).

The addition of PVPP to the two extracts of each species significantly affected (*p* < 0.05) egg hatching of *H. contortus* (Table 5) and adult *C. elegans* mortality (Table 6). In contrast, for larval exsheathment, the results seem to indicate that incubation of extracts with PVPP did not change the exsheathment process when compared to non-treated extracts (*p *> 0.05) (Fig. 1).

**Table 5:** Inhibition of egg hatching after 48 h incubation with extracts treated or not treated with polyvinyl polypyrrolidone (PVPP) for *Acacia raddiana* aqueous and acetonic extracts at a concentration of 5 mg/mL.

Extracts	Control	without PVPP (%)	with PVPP (%)
aqueous	6.2 ± 1.7	89.2 ± 6.5^a^	2.4 ± 2.9^b^
acetonic		98.6 ± 2.7^a^	3.1 ± 3.4^b^

(a,b): significant difference (*p* < 0.001) between columns.

**Table 6:** Adult *Caenorhabditis elegans* mortality percentage, after incubation for 20 h with *Acacia raddiana* and *Acacia nilotica* aqueous and acetonic extracts previously treated or not with polyvinyl polypyrrolidone (PVPP).

Extract [] 10 mg/mL	Control	Without PVPP (%)	with PVPP (%)
*A. raddiana* aqueous	0.69 ^a^	100.00 ^b^	14.91 ^c^
*A. raddiana* acetonic	0.96 ^a^	100.00 ^b^	13.26 ^c^
*A. nilotica* aqueous	0.69 ^a^	94.97 ^b^	0.00 ^ac^
*A. nilotica* acetonic	0.96 ^a^	85.89 ^b^	4.31 ^ac^

(a,b,c) = significant difference (*p* < 0.05) between values in rows.

## Discussion

The use of tannin-rich plants as an alternative treatment to chemical anthelmintics is one approach that could reduce the development of parasite resistance [32,34,57]. The objective of this study was to evaluate and compare the *in vitro* ovicidal and larvicidal efficacy of aqueous and acetonic leaf extracts of *A. nilotica* and *A. raddiana* against *H. contortus*, because these two *Acacia* species are widely used by Sahelian breeders in ethnoveterinary medicine. According to the literature, many species of *Acacia* have been reported to have anthelmintic activities: the leaves of *Acacia cyanophylla* [1], *Acacia karoo* [34], *Acacia nilotica* [34,45], *Acacia pennatula* [2,3] and *Acacia polyancatha* [42], and the bark of *Acacia mangium* [54] and *Acacia* *mearrsii* [69].

In our studies, three *in vitro* assays were performed with two extracts (aqueous and acetonic extracts) at different concentrations. These types of extracts have been commonly used in many *in vitro* tests. Moreover, solvents and protocols used for extraction caused variations in concentrations and the classes of metabolites present in extracts [40].

The quantification of tannins revealed that *A. nilotica* was 18 times richer in CT than *A. raddiana*. However, *A. raddiana* was seven times richer in TT and five times richer in TP than *A. nilotica*. According to Hoste et al. [27], tannin-rich plants could interact with the proteins of the cuticle, oral cavity, oesophagus, cloaca and vulva of nematodes, modifying their chemical and physical properties. The anthelmintic effects of tannins may be attributed to their capacity to bind free protein available in the tubes for larval nutrition, and this reduced nutrient availability could therefore have resulted in larval starvation or decrease in gastrointestinal metabolism directly through the inhibition of oxidative phosphorylation, causing larval death [7].

Our studies conducted *in vitro* with *A. nilotica* and *A. raddiana* extracts showed the inhibition action on egg hatching and larval exsheathment of *H. contortus* and mortality of *C. elegans* adults. Extracts acted in a dose-dependent manner and their efficacy differed depending on the organ. This anthelmintic efficacy may be attributed to an individual or a combined effect of the bioactive compounds.

In the EHA, *A. nilotica* extracts were not effective compared to *A. raddiana*. *A. nilotica* inhibited 22% of egg hatching for the most effective concentration (5 mg/mL) with IC_50_>5 mg/mL. However, the two extracts of *A. raddiana* presented a high ovicidal activity (more than 90% at 5 mg/mL) with different concentrations of inhibition: IC_50_ = 0.68 mg/mL for acetonic extract and IC_50_ = 1.36 mg/mL for aqueous extract. Acetonic extracts were more ovicidal than aqueous extracts, but no significant difference was recorded between the extracts in our study. Thus, the results obtained for *A. nilotica* contrasted with those obtained by Eguale et al. [24] who reported significant egg hatching inhibition for aqueous extracts for the same plant. Likewise, Badar et al. [12] found that crude aqueous methanol extracts of *A. nilotica* bark (LC_50_ = 0.201 mg/mL) had higher inhibitory effects compared with leaves (LC_50_ = 0.769 mg/mL). Other results have confirmed the anthelmintic effect of different *Acacia* species on EHA. Thus, Oliveira [54] reported IC_50_ = 1.35 mg/mL and 4.66 mg/mL with *A. mangium* extract on two *H. contortus* (White River: a strain resistant to ivermectin, benzimidazole and closantel; and the Juan strain: susceptible to all synthetic anthelmintics, respectively). Secondary plant metabolites (1) might bind the lipoproteins responsible for eggshell membrane permeability [59], and (2) tannins might inactivate enzymes responsible for the hatching process [43].

In the LEIA, both *Acacia* extracts were effective against larval exsheathment. *A. nilotica* revealed the lowest concentration of inhibition (IC_50_ = 0.195 mg/mL) for the aqueous extract compared to the aqueous extract of *A. raddiana* (IC_50_ = 0.331 mg/mL). However, the acetonic extract of *A. nilotica* was numerically higher compared to *A. raddiana*. Moreover, all extracts at 600 and 1200 μg/mL blocked the exsheathment process. Previous *in vitro* results indicated that extracts of various tannin-rich woody plants inhibited the exsheathment of nematode L_3_ [13]. Alonso-Diaz et al. [2,3] tested *Acacia pennatula* and *A. gaumeri* extracts and found an inhibition of 51% at 1200 μg/mL, 93.5% at 600 μg/mL, and a total exsheathment process after 70 min at 1.2 mg/mL, respectively. The exsheathment process in trichostrongyle nematodes is a crucial step that represents the transition from the free-living to the parasitic stages.

In the AMT, the free-living soil nematode *C. elegans* was used to test the efficacy of the extracts. *C. elegans* is a system to screen products for their potential anthelmintic effect against small ruminant gastrointestinal nematodes, including *H. contortus* [36]. The two *Acacia* extracts tested were effective against adult *C. elegans* and their lethal concentrations were less than LC_50_ < 1.5 mg/mL, except for the acetonic extract of *A. nilotica* (LC_50_ = 5.4 mg/mL). For the same concentration (2.5 mg/mL), the acetonic extract of *A. raddiana* revealed 100% adult mortality compared to 19% for *A. nilotica*. However, their two aqueous extracts revealed the same percentage of mortality (80.32 and 81.78%, respectively). In general, *A. raddiana* was more larvicidal than *A. nilotica*. *A. raddiana* contained high total tannins (TT). Plant extracts containing hydrolysable tannins (HT) such as gallic and/or ellagic acid or containing prodelphinidin CT (with gallic acid units) had higher levels of anthelmintic activity *in vitro* than the proanthocyanidin CT, which lack gallic acid units [17]. According to Katiki et al. [37] plants containing both CT and HT of the gallotannin and ellagitannin types were more lethal to *C. elegans* than plants containing CT lacking gallic acid units.

The results with PVPP for all assays showed that both extracts of *Acacia* species may have variability in the roles of tannins for EHA, LEIA and AMT. For our results, the addition of PVPP in *Acacia* extracts was associated with a significant decrease (restoration of control values) of EHA and AMT, but, did not seem to influence LEIA.

In the AMT, tannins and the associated metabolites seemed responsible for the mortality of adult *C. elegans* for *A. raddiana* extracts compared to *A. nilotica* extracts. Thus, tannins would be responsible for the efficacy of the extracts for the inhibition of egg hatching, but were not the only metabolites involved in adult mortality for *A. raddiana* extracts. These results suggest that a possible synergistic relationship of tannins and other compounds may be involved in killing the worms. For tannins, including condensed tannins, their anthelmintic activities have been reported by Athanasiadou et al. [8], Paolini et al. [57], Min et al. [48], Hoste et al. [27] and Gertrude et al. [25]. Tannins would appear to be able to bind to proteins and glycoproteins of the adult cuticle (a structure rich in proline and hydroxyproline), to the enzymes secreted by worms and involved in various essential functions [27], or to interact with their digestive epithelium to inhibit nutrition and cause the death of the parasite. Similar deductions were made with aqueous and ethanolic extracts of *Moringa oleifera* [25], aqueous extracts of *Anogeissus leiocarpus* and *Daniellia oliveri* [32], *Leucaena leucocephala* and *Gliricidia sepium* [33].In our study, the inefficacy of PVPP to restore control values of larval exsheathment suggested that tannins were not the metabolites responsible for anthelmintic activity on *H. contortus*. According to Chan-Pérez et al. [21] and Vargas-Magana [68], tannins were not the sole secondary plant metabolites responsible for the AH effects. Our results contrasted with many previous studies that suspected tannins of being responsive to inhibit exsheathment [2,43,44,52]. Thus, Brunet et al. [18] and Hoste et al. [28] observed a structural lesion on *H. contortus* L_3_ larvae (*in vitro* and *in vivo*) in contact with sainfoin extracts, a tannin-rich plant. Oliveira et al. [53], showed the inhibition of larval exsheathment in *H. contortus* subject to *A. mangium* extract. Son-de Fernex et al. [64] observed an inhibition effect on exsheathment of *H. contortus* larvae subjected to five plants containing CTs. Consequently, we think that other metabolites besides tannins would be able to affect the exsheathment kinetics of L_3_ larvae by binding to proteins and glycoproteins of the sheath to prevent the formation of the indented ring in the anterior part.

The work of Barrau et al. [14] and Ayers et al. [9] showed that flavonoids could play an essential role in the anthelmintic activity of some plants. Thus, the sheath of infective larvae of parasitic nematodes would be the target of flavonoids [10,19] as well as polyphenols [4]. Bizimenyera et al. [16] showed that polyphenols could have anthelmintic activities on nematodes. These chemical groups may be able to inhibit the secretion of proteases and acetyl-cholinesterase fluid by the glandular cells of the larvae to thereby prevent digestion and separation of the cap from the rest of the sheath.

However, other bioactive compounds and secondary plant metabolites could interact with multiple molecular targets on the various developmental stages of the parasite. Nandi et al. [50] showed that saponins have the ability to generate ions and cause lipid peroxidation of egg membranes, as well as to cause damage at the larval cuticle to inhibit parasite development. Other authors suggest that the conjugated unsaturated system of the saponins is involved in producing their damaging effect, probably resulting in free radicals, which induce membrane damage through peroxidation in nematodes [62].

*A. nilotica* and *A. raddiana* extracts both have *in vitro* anthelmintic activities against *H. contortus* and *C. elegans*. The two species of *Acacia* had completely different tannin contents. *A. nilotica*, rich in CT, was (1) not effective in inhibiting egg hatching; (2) partially effective for adult mortality; and (3) effective in inhibiting larval exsheathment. However, *A. raddiana*, rich in TT and TP, was highly effective in the three assays. Thus, in our study, TT-rich plants showed higher and more diverse anthelminthic activities against *H. contortus* compared to CT-rich plants. HT and CT are both found in TT. It is therefore possible that HT are responsible for the efficacy of *A. raddiana* extracts. Moreover, HT are known to show some toxicity for the ruminants, but the leaves of this plant were consumed by small ruminants in the Sahelian region of Burkina Faso. It is possible that the types and the quantity of HT contained in the leaves were not sufficient to cause ruminal toxicity but could affect the parasites. Future studies are required to ascertain which types or HT (gallotannins or ellagitannins) are contained in *A. raddiana* extract and underlie the anthelminthic activities. In general, the two species of *Acacia* used in our study were effective against *H. contortus*. Therefore, the association of these *Acacia* in the treatment of gastrointestinal parasites in a traditional environment might be a valuable approach. However, it would be necessary to conduct *in vivo* parasitological studies to consider the metabolism of extracts in the digestive tract of ruminants.

## Conflict of interest

The authors declare that they have no competing interests.
